# Establishment of an amino acid metabolism related signature for prognostic and therapeutic sensitivity prediction in breast cancer by machine learning

**DOI:** 10.1371/journal.pone.0340586

**Published:** 2026-01-09

**Authors:** Xinrui Zhao, Jie Li, Nan Hu, Xiaoming Wu, Junbo Duan

**Affiliations:** 1 Key Laboratory of Biomedical Information Engineering of Ministry of Education, Department of Biomedical Engineering, School of Life Science and Technology, Xi’an Jiaotong University, Xi’an, China; 2 First Affiliated Hospital, Xi’an Jiaotong University, Xi’an, China; University of Helsinki: Helsingin Yliopisto, FINLAND

## Abstract

Amino acid metabolism plays a critical role in tumor growth and immune regulation, yet its comprehensive function in breast cancer remains underexplored. We developed an amino acid metabolism–related gene signature (AAMRGS) to predict prognosis and therapeutic response in breast cancer. The AAMRGS was constructed using a machine-learning framework integrating ten algorithms and validated across multiple independent cohorts. It served as an independent prognostic factor and outperformed existing amino acid metabolism–related signatures and clinical variables. Moreover, the prognostic utility of AAMRGS was further validated across pan-cancer datasets, and an AAMRGS-based nomogram was constructed to facilitate clinical application. Functional enrichment and protein–protein interaction analyses revealed that AAMRGS genes were primarily involved in metabolic reprogramming and cell proliferation. Experimental validation confirmed the downregulation of key genes such as *SAV1* and *IGF2R* in breast cancer cells. Integrative analyses revealed that the high-AAMRGS subgroup exhibited a greater copy number variation burden, higher tumor mutation burden (TMB), enrichment of immunosuppressive cell populations, and increased sensitivity to most chemotherapeutic drugs. In contrast, the low-AAMRGS subgroup displayed higher immune scores, stronger immune activation, enrichment of anti-tumor immune cells, and greater responsiveness to immunotherapy. Collectively, our findings establish AAMRGS as a reliable prognostic signature and a potential tool to guide individualized therapeutic strategies for breast cancer patients.

## 1. Introduction

Breast cancer remains one of the most prevalent malignancies affecting women worldwide. In 2022, it accounted for approximately 31% of all newly diagnosed cancers and 15% of cancer-related deaths among women in the United States, according to the American Cancer Society [1,2]. Although, diverse types of biomarkers and potential therapeutic targets for breast cancer have been developed in recent years [3–5], their clinical performance remains suboptimal [6,7]. Notable examples include Oncotype DX, ER/PR/HER2, and Ki-67, which have been used to assess prognosis and predict chemotherapy responses. Nevertheless, the effects of novel biomarkers on diagnosis and prognosis remain unsatisfactory. For instance, Oncotype DX is validated for early-stage ER+ /HER2−, node-negative disease, with limited evidence supporting its utility in other subtypes [8,9]. Similarly, the widely used IHC-based classification relying on ER, PR, HER2, and Ki-67 suffers from poor reproducibility, often leading to over- or undertreatment [10]. Thus, it is crucial to recognize new biological markers and construct reliable prognostic models for outcome prediction and treatment.

Metabolic reprogramming is recognized as a key characteristic of cancer [11]. Tumor cells have a high energy demand for supporting proliferation and survival [12]. Apart from the Warburg effect, amino acid metabolism participates in tumor growth and proliferation, some of which provide a fundamental role in cellular redox, genetic and epigenetic status [13–15]. A feedforward loop involving oncogenic *MYC*, *SLC7A5*, and *SLC43A1* has been shown to promote amino acid transport and tumorigenesis [16]. Moreover, a study has shown that amino acid is a part of the tumoral metabolic network between stromal and tumor cells [17]. In the immune microenvironment, amino acid metabolism holds pivotal functions in the metabolic rewiring of immune cells and supports kinds of immune cell functions [18]. Glutamine is an important nutrient with surprising roles in sustaining the biological hallmarks of cancer [19]. Inhibiting glutamine metabolism suppresses oxidative and glycolytic pathways in cancer cells while enhancing oxidative metabolism and activation in effector T cells, revealing a “metabolic checkpoint” that may be exploited for immunotherapy [20]. Thus, these findings underscore the therapeutic potential of targeting amino acid metabolism [21–23].

In breast cancer, amino acid metabolic pathways are altered, suggesting its potential role in proliferation and progression [24]. Overexpression of transporters such as *SLC7A5*, *SLC1A5*, and *SLC6A14* accelerates the metabolism of glutamine and promotes tumor growth in triple-negative breast cancer (TNBC) [24,25]. Enhanced serine synthesis has also been linked to increased cell proliferation, highlighting the significance of the serine synthesis pathway [26,27]. Furthermore, basal-like breast cancer exhibits an inverse relationship between glutamine metabolism-related genes and T cell-mediated cytotoxicity, and increasing glutamine metabolism was associated with poor survival [28]. Collectively, these findings suggest that genes involved in amino acid metabolism may hold prognostic values and represent potential therapeutic targets in breast cancer.

In this study, we aimed to develop an amino acid metabolism–related gene signature (AAMRGS) to predict prognosis and therapeutic response in breast cancer. Using an integrated machine-learning framework, we identified the optimal AAMRGS and validated its performance in external and pan-cancer datasets. Then, we compared AAMRGS with other signature-related amino acid metabolism and created an individualized nomogram that integrated AAMRGS and clinical features. Furthermore, we explored the molecular subtypes, genomic variation, immune landscape, and sensitivity to chemotherapy and immunotherapy associated with AAMRGS-defined subgroups, providing insights into metabolic heterogeneity. We established the AAMRGS as a prognostic tool, which might provide treatment guidance for individualized therapy in breast cancer.

## 2. Methods

### 2.1. Data collection and process

Breast cancer samples from The Cancer Genome Atlas (TCGA) datasets, clinical information, and pan-cancer data were obtained from XENA [29]. TCGA-BRCA dataset includes RNA-seq data for 1052 breast cancer patients and 30691 genes after data pre-processed. Raw counts were converted into TPM values and subsequently log_2_ transformed. The Molecular Taxonomy of Breast Cancer International Consortium (METABRIC) dataset was downloaded from cBioPortal database [30]. The METABRIC dataset comprises microarray data from 1,904 breast cancer patients, with 24166 genes after data pre-processed. The expression matrix was already normalized by the data provider. GSE96058 was derived from the Gene Expression Omnibus database (GEO) [31], included RNA-seq data from 3409 breast cancer patients, providing gene expression profiles for 28629 genes after data pre-processed (log2-transformed FPKM values). In this study, TCGA was used as the training dataset, while METABRIC, GSE96058 and GSE20685 were employed as independent validation datasets. To ensure comparability across datasets, only tumor samples with overall survival time > 30 days were included. Given the inherent differences in data derived from distinct platforms (RNA-seq for TCGA and GSE96058, and microarray for METABRIC), we did not integrate these datasets into a single large dataset but instead treated them as independent cohorts to assess the robustness and generalizability of the developed AAMRGS. The search term “amino acid metabolism related genes” was used for query in GeneCards database and genes correlated with the relevance score over 7 (S1 Table) were downloaded from GeneCards [32] which contained 16111 genes. Both Kaplan–Meier survival analysis and univariate Cox regression in each dataset (TCGA-BRCA, METABRIC, and GSE96058). Genes with p < 0.05 in both KM and Cox regression were considered prognostic candidates.

### 2.2. Machine learning to develop the prognostic signature based on AAMRG

The R package “tinyarray” was utilized for conducting the Kaplan-Meier analysis and univariate Cox regression to determine AAMRG that were correlated with overall survival (OS) in both the training and external validation datasets. The comprehensive framework integrated ten distinct machine learning algorithms for screening variables and developing the prognostic signature, following the 101 algorithms strategy described previously (PMID: 35145098) [33]. The algorithms included RSF (random survival forest), Enet (elastic network), Lasso, Ridge, stepwise Cox, CoxBoost, plsRcox (partial least squares regression for Cox), SuperPC (supervised principal components), GBM (generalized boosted regression modeling), and survival-SVM (survival support vector machine). Based on these ten algorithms, 101 possible combinations were generated according to the original framework. In our study, we applied a minimum threshold of five selected variables; algorithm combinations yielding fewer than five variables were considered uninformative and excluded from further analysis. Consequently, 98 algorithms were retained and used for constructing and validating the AAMRGS.

The Lasso, Ridge, and Elastic Net methods were applied within the Cox regression framework to select features for survival analysis, and the resulting coefficients were used to calculate the risk scores for each patient. We used the “glmnet” package to implement the Elastic Net model within a Cox regression framework. Specifically, the family = “cox” parameter ensures that the model predicts risk scores rather than survival times. The cv.glmnet function performs 10-fold cross-validation to select the optimal regularization parameters (lambda), and the glmnet function trains the final model using these parameters. The Lasso and Ridge models were treated as special cases of the Elastic Net, implemented by calling the RunEnet function with alpha = 1 and alpha = 0, respectively. The stepwise Cox regression was performed by “survival” package. The direction parameter specifies whether the process is forward, backward, or both. This method used the AIC (Akaike information criterion) to add or remove variables.

We used the “survivalsvm” package to implement the SurvivalSVM model. This model is specifically designed for survival analysis, the default optimization method (opt.meth = “ipop”) was used, and gamma.mu = 1. The CoxBoost model was implemented by “CoxBoost” package, we used 10-fold cross-validation to select the optimal number of steps and the penalty parameter (optimCoxBoostPenalty). The SuperPC model was implemented using the “ superpc “ package. We adapted it for survival analysis by specifying type = ‘survival’ and used 10-fold cross-validation to select the optimal threshold. The plsRcox model was performed by “plsRcox” package, and 10 cross-validation was used to select the optimal number of components. The RSF model was implemented by the “randomForestSRC” package. The internal parameters (ntree & nodesize) control the tree-building process. We used “superpc” package to develop GBM model and 10-fold cross-validation to select the optimal number of trees.

The average and the variance of C-index (concordance index) for each method were calculated. The final prognostic signature was identified by setting a range of 5–15 for the number of features. The details C-index of these method are outlined in S2 Table. Model performance was evaluated based on mean C-index, variance, and the number of selected features. The signature associated with highest average C-index and lowest variance was then chosen as the optimal signature.

The AAMRGS score for every patient was calculated as a linear model: AAMRGS score = Coef ^T^ * Exp.

Where Exp is a column vector that stores the expression values of the AAMRG and Coef is also a column vector of the same length as Exp that stores the regression coefficients calculated by multivariate Cox regression analysis. Afterward, patients were categorized into two risk subgroups according to the median value of the AAMRGS score. Patients were stratified into high- and low-risk groups based on the median AAMRGS score to maintain balanced subgroup sizes. This approach minimizes class imbalance, ensuring robust statistical comparisons.

Kaplan-Meier survival analysis was performed by R packages “survival” and “survminer”. The capability of the AAMGRS model was detected using time-ROC (time-dependent receiver operating characteristic) curves with R package “timeROC”. The AAMRGS was compared with other published amino acid metabolism-related signatures (S3 Table), and further validated in pan-caner datasets.

### 2.3. Construction of nomogram prognosis prediction model

A personalized prognostic nomogram was developed using the R package “rms” in training dataset, as described in previous bioinformatics studies [34,35]. Using the R package “rms”, we constructed the calibration plot to estimate the discrepancy between the predicted values and the actual observed values. The “ggDCA” R package was employed to generate the decision curve analysis (DCA), accessing the nomogram’s net benefit. Continuous Net Reclassification Index (NRI) and Integrated Discrimination Improvement (IDI) values were computed using the “survIDINRI” R package based on time-dependent Cox models.

### 2.4. Exploration of AAMRGS subgroups based on genomics data

Somatic mutation information was downloaded from TCGA database and to assess the tumor mutation burden (TMB). The TMB was determined by the sum of mutations per megabase (mut/Mb) using the ‘maftools’ R package [36]. Copy number variation (CNV) data were obtained via “TCGAbiolinks” and analyzed using GISTIC2 to identify amplifications and deletions for each sample. The burden of copy number variation was calculated by totaling the number of genes displaying amplification or deletion.

### 2.5. Functional enrichment analysis and protein-protein interaction (PPI) network

The AimGO2 (https://amigo.geneontology.org/amigo) and KOBAS (http://bioinfo.org/kobas/) were used to perform enrichment analysis for AAMRG that had a relevance score of 7 or higher. To identify the diverse biological processes across the two risk subgroups, enrichment analysis of biological processes in the Gene Ontology (GO) and pathways in the Kyoto Encyclopedia of Genes and Genomes (KEGG) [37,38] was performed using the “gene set variation analysis (GSVA)” R package” [39].

The protein-protein interaction (PPI) network of key genes was generated by an online tool, GeneMANIA (http://genemania.org/). The key genes and co-expression genes were further to perform enrichment analysis by Metascape (https://metascape.org/gp/index.html).

### 2.6. Correlations between the stemness index and AAMRGS

Cancer progression is characterized by the loss of a differentiated phenotype and undifferentiated tumors are more caused by disease progression and poor prognosis. To estimate the stemness index, we utilized gene expression data to calculate the mRNAsi, which stands for the stemness index according to mRNA expression [40], and ranges from low (zero) to high (one). The stemness was calculated using R package “GSVA” and the collection of stemness signature was sourced from a prior study [41]. We also implemented an alternative calculation method based on the approach outlined in Reference 36.

### 2.7. Exploration of infiltration of immune cells, immune subtypes and tumor microenvironment at two risk subgroups

The single sample gene set enrichment analysis (ssGSEA) was carried out by R package “GSVA” that along with CIBERSORT algorithm [42], identified the infiltrate levels of immune cell enrichment in the tumor microenvironment of each breast cancer sample. The ESTIMATE algorithm was utilized to estimate both the tumor purity (tumor purity = cos (0.6049872018 + 0.0001467884*ESTIMATEScore)) and immune score [43,44]. The tumor microenvironment subtypes were subsetted from the research by Alexander Bagaev [45]. These tumor microenvironment subtypes include IE (Immune-enriched, non-fibrotic), IE/F (Immune-enriched, Fibrotic), D (Immune desert), and F(Fibrotic). Six immune subtypes (C1-C6) were determined using the “ImmuneSubtypeClassifier” R package [46]. The immune subtypes were C1 (Wound Healing), C2 (IFN-γ Dominant), C3 (Inflammatory), C4 (Lymphocyte Depleted), C5 (Immuno-logically Quiet), C6 (TGF-β Dominant). TIP (http://biocc.hrbmu.edu.cn/TIP/) was used to analyze the activity of anticancer immunity scores on each sample [47].

### 2.8. Correlations between molecular subtypes and subgroups

The intrinsic molecular subtypes of breast cancer patients (Normal-like, Basal-like, Her2-enriched, LumA, and Lum B) were classified by the PAM50 function [48] that was offered by the R package “genefu”.

### 2.9. Drug sensitivity and immunotherapy sensitivity analysis

The package “pRRophetic” was applied to compare the sensitivity to chemotherapeutic drugs in two risk subgroups, as described in previous bioinformatics studies [49,50]. The analysis of response to immunotherapeutic sensitivity was performed by TIDE, and the immunophenoscore (IPS) file of breast cancer was downloaded from TCIA (https://tcia.at/), as described in previously [51,52].

### 2.10. Cell lines and quantitative real-time PCR(RT-qPCR)

The MCF-10A normal human mammary epithelial cell line and MCF-7 and MDA-MB-231 human breast cancer cell lines were obtained from an authenticated cell bank. All cells were cultured at 37°C in a humidified incubator containing 5% CO₂.

Total RNA was extracted using the RNAex Pro Reagent (AG, Hunan, China; Cat. No. AG21101) according to the manufacturer’s protocol. Complementary DNA (cDNA) was synthesized using the Evo M-MLV RT Premix (AG, Hunan, China; Cat. No. AG11706). Quantitative real-time PCR (qRT-PCR) was performed using the SYBR Green Pro Taq HS Premix IV (AG, Hunan, China; Cat. No. AG11746) on a QuantStudio™ 3 Real-Time PCR System (Thermo Fisher Scientific, USA).

The 2 ⁻ ΔΔCt method was applied to calculate relative gene expression, with GAPDH serving as the internal control. Primer sequences used in this study were as follows:

*GAPDH* (Forward) 5’-CACCCACTCCTCCACCTTTGAC-3’
*GAPDH* (Reverse) 5’-GTCCACCACCCTGTTGCTGTAG-3’
*SAV1* (Forward) 5’- CACGAGCCCCTGTGAAATAT -3’
*SAV1* (Reverse) 5’-TTAGCATTCCCTGGTATGTATCCA-3’
*IGF2R* (Forward) 5’- AGGTGAAGCCCAACGATCAG-3’
*IGF2R* (Reverse) 5’- GACATCGAGATCGCCGTCTT-3’

### 2.11. Statistical analysis

Statistical analyses were performed using R version 4.3.2, with all parameters set to their default values. The prognostic value was analyzed by Kaplan-Meier analysis and COX analysis. The Wilcoxon test was employed to evaluate the statistical discrepancies in two subgroups. For multi-group comparisons, Kruskal-Wallis test was first performed to assess overall statistical significant. If the result was significant, the Wilcoxon test with a Benjamini-Hochberg (BH) adjustment for multiple comparisons was used to evaluate pairwise statistical significance between subgroups. S4 Table shows the corresponding R codes in this study. Statistical analysis of cell line experiments was performed using one-way ANOVA. Results are presented as the mean ± standard deviation (SD) and visualized using GraphPad Prism 10. The significant difference was considered when p < 0.05, and * for p ≤ 0.05, ** for p ≤ 0.01, and *** for p ≤ 0.001.

## 3. Results

### 3.1. Identification of amino acid metabolism-related prognostic genes and development of AAMRGS by machine learning

**Fig 1:**
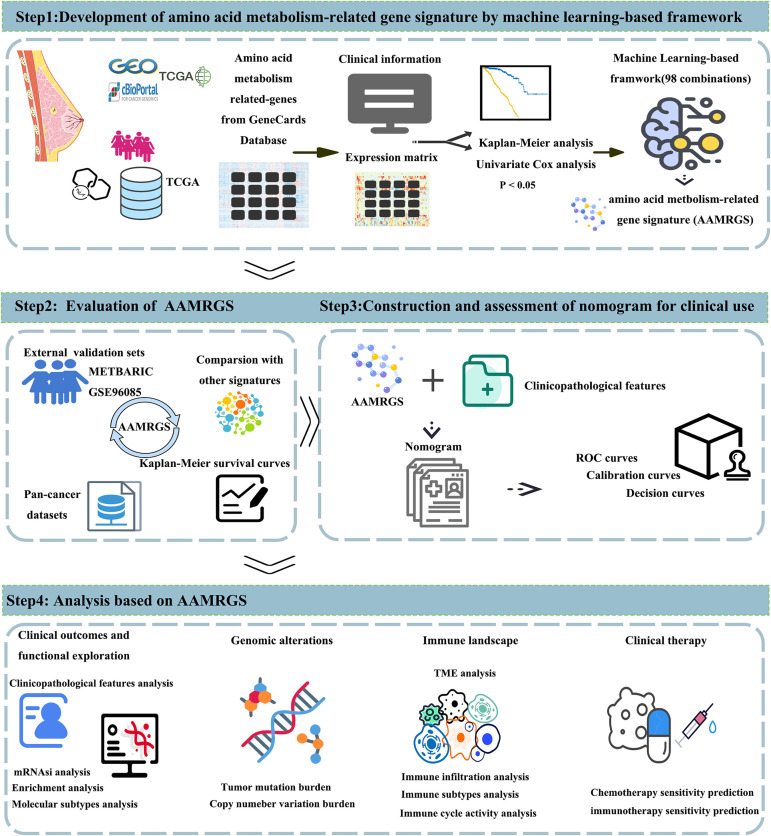
Overall workflow. The first step is to identify and develop the AAMRGS by machine learning-based framework. The second step is to evaluate the AAMRGS in external validation datasets and pan-cancer datasets. And comparison of AAMRGS with other amino acid metabolism-related signatures. The third step is to construct a nomogram integrating AAMRGS with clinical features. The fourth step is to analyze the differences in the two risk subgroups in clinicopathological features, genomic alterations, immune landscape, and therapeutic sensitivity.

**Fig 2:**
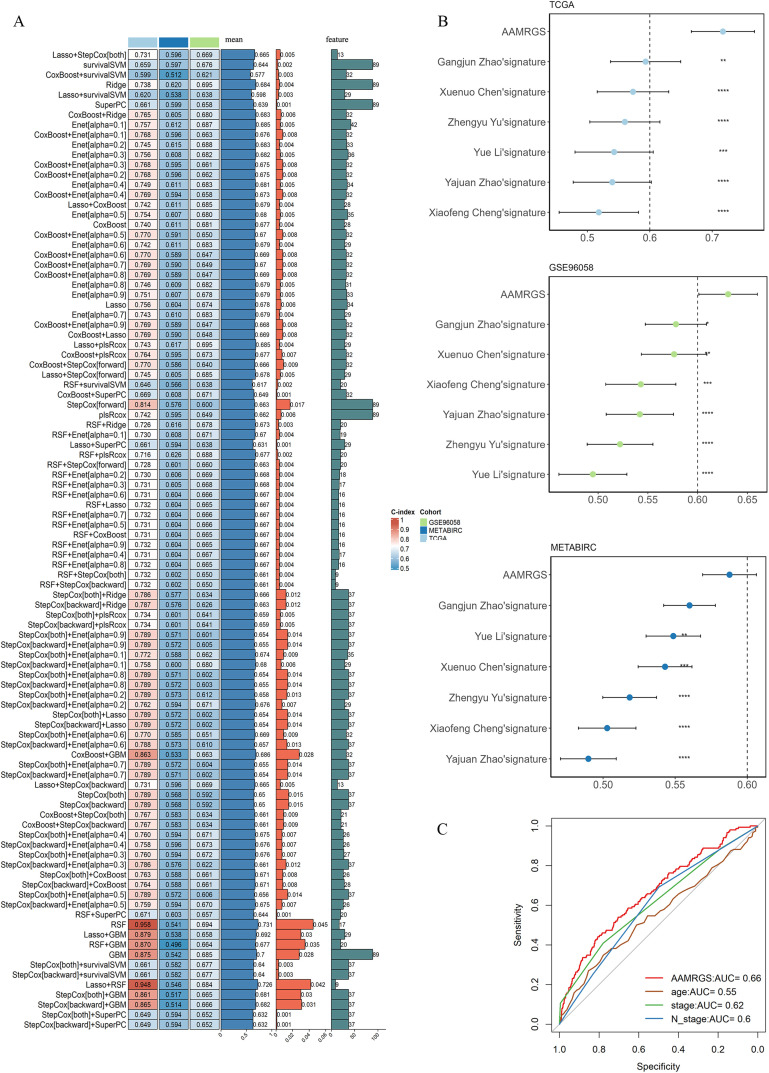
Development and evaluation of AAMRGS by machine learning framework. **(A)** Heatmap of 98 machine learning-based prognostic models in all datasets and their average C-index values. **(B)** Forest plots of AAMRGS and published amino acid metabolism-related signatures in TCGA, METABRIC, and GSE96058 datasets. **(C)** The ROC curve showed AAMRGS and other clinical features. Levels of statistical significance were defined as *p ≤ 0.05, **p ≤ 0.01, and ***p ≤ 0.001.

Fig 1 provides the overall workflow of our study. To isolate signature genes associated with amino acid metabolism, we first selected AAMRG with a relevance score ≥7 from the GeneCards database as candidate genes. These genes enriched in pathways associated with amino acid biosynthesis, energy metabolism, and cell signaling (S5 Table). Subsequently, 89 genes were identified as significantly associated with overall survival in both training (TCGA) and external validation datasets (METABRIC & GSE96058) based on Kaplan-Meier analysis and univariate cox regression (S1, S2 Figs, S6 Table). The AAMRGS was constructed from these 89 candidate genes using a machine learning integration framework. A total of 98 prognostic prediction signatures were obtained. Considering the simplicity, accuracy, and robustness of our signature, the Lasso+StepCox[both] was ultimately determined. Although both linear and nonlinear models were evaluated, Lasso+StepCox[both] demonstrated superior stability, consistent performance across different datasets, and high interpretability. Models with higher mean C-index and lower variance were prioritized to ensure performance in both training (TCGA) and external validation datasets (METABRIC & GSE96058). Furthermore, we limited the number of features to between 5 and 15 genes to enhance clinical feasibilty and interpretablility of the model. Among all methods with signature genes ranging from 5 to 15, the Lasso+StepCox[both] exhibited the highest average C-index value and the smallest variance (Fig 2A). AAMRGS exhibited a higher C-index value when compared to existing published signatures(Yue Li, Xuenuo Chen, Guangjun Zhao, Zhengyu Yu, Yajuan Zhao, Xiaofeng Cheng) related to AAMRG (Fig 2B). To further assess the predictive efficacy of the AAMRGS for breast cancer patient’s prognosis, receiver operating characteristic (ROC) curves suggested that the AAMRGS exhibited superior predictive performance compared with traditional clinicopathological features in breast cancer [53] (Fig 2C).

### 3.2. Prognostic evaluation of AAMRGS and construction of AAMRGS-based nomogram

**Fig 3:**
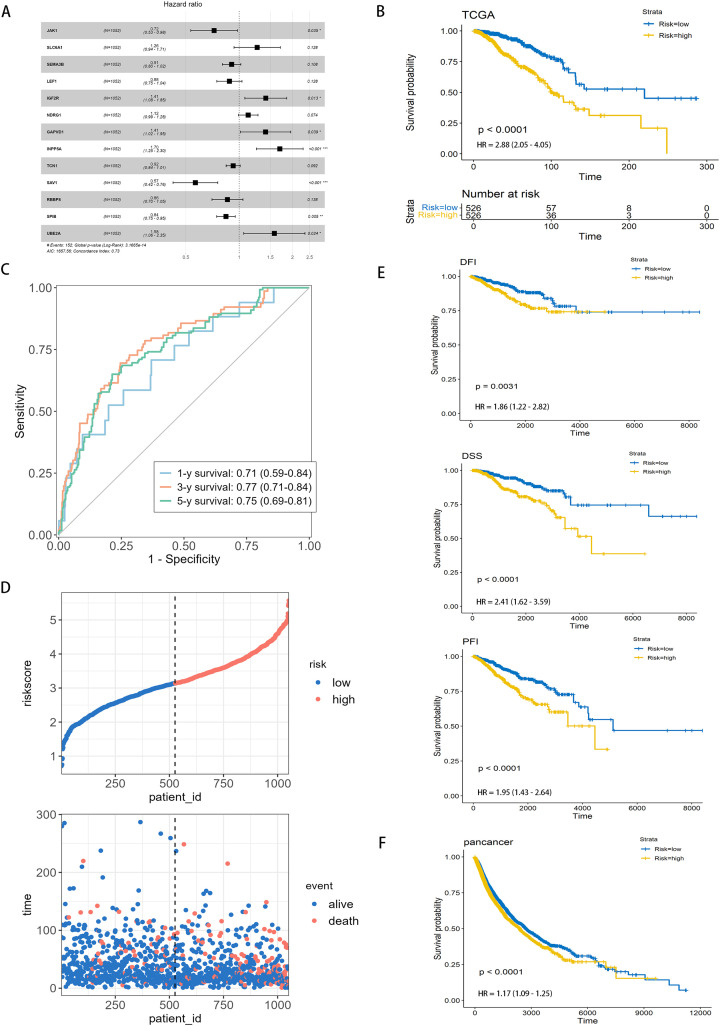
Role of AAMRGS in predicting the prognosis value. **(A)** The forest plot displayed thirteen AAMRG selected by multivariable cox (AIC) analysis, which constructed the optimal signature. **(B)** Kaplan–Meier survival analysis revealed that high-AAMRGS subgroup, denoted in yellow, exhibited inferior survival outcomes when compared to the low-AAMRGS subgroup, represented in blue. **(C)** Time-dependent ROC curves illustrated the 1-year, 3-year, and 5-year survival rates of patients, showcasing the model’s predictive accuracy over these distinct time intervals. **(D)** The top graph represents the classification of patients into high (red) and low (blue) risk groups based on risk scores, and the bottom graph represents as the patient’s risk score increased, the mortality rate also increased. **(E)** Kaplan–Meier analysis of the disease-free interval (DFI), disease-specific survival (DSS), and progression-free interval (PFI) in two risk subgroups, indicating superior outcomes for the low-AAMRGS subgroup across these critical survival metrics. **(F)** Kaplan–Meier analysis across a pan-cancer dataset showed that low-AAMRGS subgroup exhibited significantly enhanced overall survival times in comparison with the high-AAMRGS subgroup. Levels of statistical significance were defined as *p ≤ 0.05, **p ≤ 0.01, and ***p ≤ 0.001.

To assess the prognostic utility of the AAMRGS, breast cancer patients were separated into two risk subgroups based on the median of the AAMRGS score. Each patient’s AAMRGS score was generated by thirteen genes (S3A, S3B Fig, S7 Table). Among these genes, *SLC6A1, IGF2R, NDRG1*, *GAPVD1*, *INPP5A*, and *UBE2A* were risk genes with hazard ratios (HRs) > 1 (S8 Table), and higher expression levels were associated with poorer survival. Conversely, *JAK1*, *SEMA3B*, L*EF1*, *TCN1*, *SAV1, RBBPB,* and *SPIB* were protective genes with HRs < 1, and higher expression levels were associated with better survival outcomes (Fig 3A). The Kaplan-Meier survival analysis indicated that patients in the low-AAMRGS subgroup had better OS compared with those in the high-AAMRGS subgroup (p-value <0.0001; Figs 3B, S3C–S3E), confirming the prognositic value of AAMRGS. Moreover, the predictive accuracy of AAMRGS was evaluated by ROC curve analysis. The area under the ROC curve (AUC) values of the patients’ 1, 3, and 5 years survival rates were 0.71, 0.77, and 0.75 in training dataset (TCGA) (Fig 3C). Consistent results were oberseved in the external validation dataset (METABRIC & GSE96058; S3F, S3G Fig). Analysis of the AAMRGS score distribution and patient survival status revealed that as the AAMRGS score escalated, survival rate decreased correspondingly (Figs 3D, S3H, S3I). These results suggest that AAMRGS can stratify patients into distinct risk groups, which may guide treatment decisions. Specifically, patients with high-AAMRGS may benefit from more aggressive therapies, while those with low-AAMRGS may be suitable for more conservative approaches. Moreover, we found that the low-AAMRGS subgroup exhibited significantly better DSS, DFI, and PFI in comparison with the high-AAMRGS subgroup (Fig 3E). To explore the broader applicability of the AAMRGS, we further assessed its prognostic performance in pan-cancer (Fig 3F). Pan-cancer analysis indicated that AAMRGS also exhibited prognostic significance in 5 cancers, including KIRC, COAD, LGG, and SKCM (S4A Fig). These results indicate that AAMRGS is a robust and effective prognostic indicator for breast cancer, with potential applicability across multiple tumor types.

**Fig 4:**
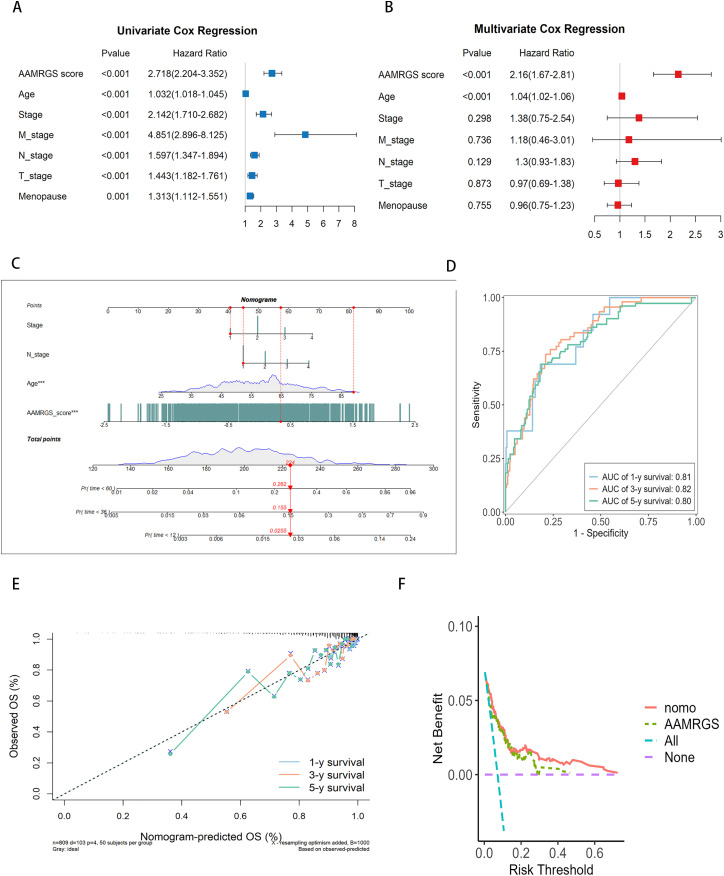
Development and assessment of AAMRGS-based nomogram. **(A-B)** Univariate (A) and multivariate (B) cox proportional hazards model facilitated the identification of independent factors predictive of prognosis. **(C)** The AAMRGS-based nomogram plot was established based on N_stage, Stage, Age, and AAMRGS. The red line was an example, the example calculation was provided: for a patient with a Stage: 1 (40 points), N_stage: 1 (44 points), Age: 90 (82 points), AAMRGS_score: 0.35 (58 points), Total points = 40 + 44 + 82 + 58 = 224, Pr (time < 60): 0.262, Pr (time < 36): 0.155, Pr (time < 12): 0.0255. **(D)** Time-dependent ROC curves were generated for the AAMRGS-based nomogram to assess patients’ 1, 3, and 5-year survival rates. **(E)** The calibration curves for AAMRGS-based nomogram were constructed to evaluate the accuracy of predicting 1-, 3-, and 5-year overall survival probabilities. **(F)** The decision curve evaluated clinical benefits of AAMRGS-based nomogram, with the risk probability displayed on the x-axis and the net benefit illustrated on the y-axis.

To explore whether the AAMRGS functions as an independent prognostic factor, we performed Cox regression analyses. The hazard ratios of AAMRGS were 2.718 (pvalue < 0.001, Fig 4A) in univariate Cox regression and 2.16 (pvalue < 0.001, Fig 4B) in multivariate Cox regression, suggesting that the AAMRGS was independently associated with patient outcomes. As previously shown in Fig 2C, we compared prognostic accuracy of AAMRGS with established clinicopathological features, including tumor stage and age (the AUCs of AAMRGS, age, stage, N_stage were 0.66, 0.55, 0.62, 0.6 respectively), indicating that AAMRGS outperformed traditional clinical factors in predicitng prognosis. Therefore, we integrated AAMRGS with clinical variables to develop a nomogram for practical application. The nomogram allows the estimation of total points corresponding to 1-, 3-, and 5-year OS (Fig 4C). Each variable, including clinical features and the AAMRGS correspond to a specific point value; the total score derived from these points corresponds to the predicted survival probability. The ROC curves revealed that nomogram exhibited good specificity and sensitivity for predicting prognosis, with AUCs of 0.81, 0.82, and 0.80 for predicting the patients’ 1-,3- and 5-year OS, respectively (Fig 4D). Consistent results were obtained in external validation set (S5A, S5B Fig). These findings indicate that integrating AAMRGS with clinical parameters substantially enhances predictive accuracy compared with AAMRGS alone. To further assess its clinical utility, calibration and decision curve analyses (DCA) were performed.The calibration curve demonstrated that the estimated values closely aligned with reference line, revealed that a high degree of concordance between predicted overall survival rates and practical observed overall survival rates (Figs 4E, S5C, S5D). The DCA revealed that the nomogram provided a greater clinical benefit than either the strategy of treating all patients or treating none (Figs 4F, S5E, S5F). We additionally evaluated whether the combined model offered superior predictive discrimination compared with the AAMRGS alone by calculating the Integrated Discrimination Improvement (IDI) and Net Reclassification Improvement (NRI) at 1-, 3-, and 5-year survival time points. As shown in S9 Table, both IDI and NRI values were positive across all time points, indicating enhanced discriminative ability and reclassification accuracy of the combined model. Specifically, the IDI values were 0.049, 0.067, and 0.091 at 1-, 3-, and 5-year survival, respectively, demonstrating a gradual improvement in discrimination over time. The corresponding NRI values were 0.568, 0.280, and 0.271 (all p < 0.01). The IDI curves (S5G Fig) visually confirmed that the combined model achieved a higher cumulative probability distribution for event prediction compared with the AAMRGS model, as reflected by the expanded red area. These results demonstrated that AAMRGS-based nomogram was accurate and stable for breast cancer. The AAMRGS-based nomogram may provide a practical tool for clinicians to estimate individual patient risk and suitable treatment plans accordingly.

### 3.3. The correlation of AAMRGS with clinical characteristics, molecular subtypes and pathways

**Fig 5:**
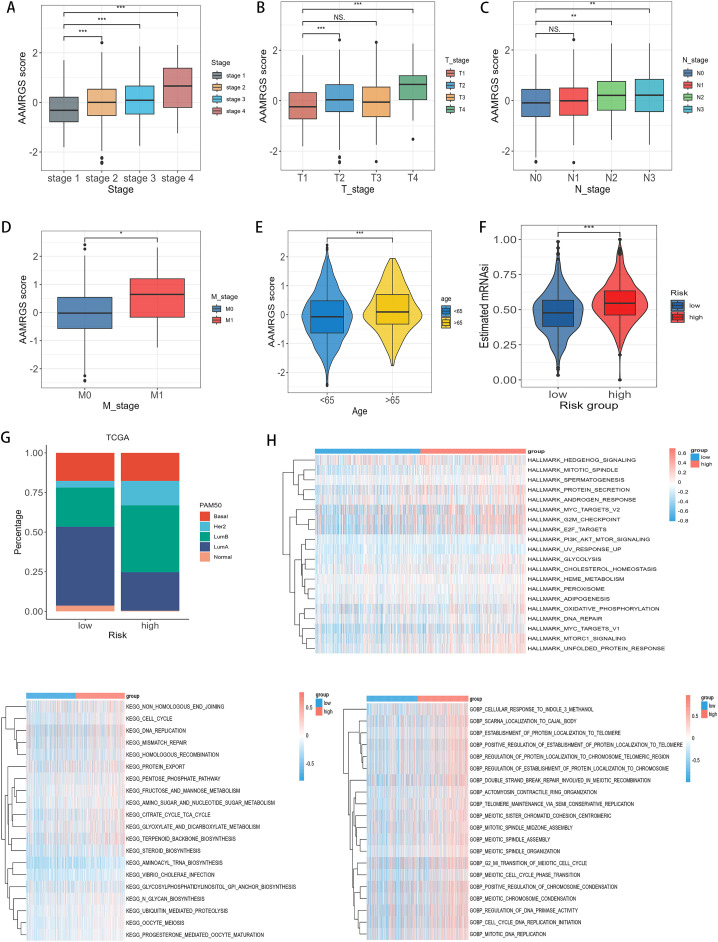
Clinicopathological characteristics and pathway characteristics among AAMRGS-based subgroups. **(A-E)** The boxplot showed the distribution of AAMRGS score among different clinicopathological features: tumor stage **(A)**, tumor T_stage **(B)**, tumor N_stage **(C)**, tumor M_stage **(D)**, age **(E)**. **(F)** The prediction of stemness index in two risk subgroups. **(G)** Distribution of PAM50 molecular subtypes in two risk subgroups. **(H)** The hallmark (e.g., Hedgehog signaling, Mitotic spindle, MYC target, G2M checkpoint, Glycolysis, Oxidative phosphorylation), KEGG pathways (e.g., Nonhomologous end joining, Cell cycle, DNA replication, Citrate cycle, Pentose phosphate pathway), and GO BP (e.g., Meiotic cell cycle phase transition, Regulation of DNA primase activity, Cell cycle, DNA replication initiation, Mitotic DNA replication) were enriched in two risk subgroups. For two-group comparisons (Wilcoxon rank-sum test) and multi-group comparisons (Kruskal-Wallis rank-sum test followed by pairwise Wilcoxon tests with BH adjustment). Levels of statistical significance (wilcox.test) were defined as NS > 0.05, * p ≤ 0.05, ** p ≤ 0.01, and *** p ≤ 0.001.

We noticed that the AAMRGS score significantly increased with advancing clinical stage (Fig 5A). Further analysis detected that AAMRGS score varied notably across different T stages (Fig 5B), N stages (Fig 5C), and M stages (Fig 5D). Moreover, our findings demonstrated that the AAMRGS score significantly differed between patients aged < 65 and those aged > 65 years (Fig 5E). To assess the prognostic robustness of the AAMRGS across different clinical subgroups, we performed Kaplan–Meier survival analyses stratified by clinical stage, T stage, N stage, M stage, and age. As shown in S6 Fig, patients in the high-AAMRGS subgroup consistently demonstrated poorer overall survival compared to those in the low-AAMRGS subgroup across most clinical strata, indicating that the prognostic value of the AAMRGS is independent of traditional clinical variables.

The stemness index has been asscociated with recurrence, proliferation, and drug resistance. Therefore, we examined the mRNA stemness index (mRNAsi) in the two subgroups. As shown in Figs 5F and S7A, our analysis revealed that high-AAMRGS subgroup exhibited a high level of mRNAsi compared to low-AAMRGS subgroup. Furthermore, a positive association between the AAMRGS score and mRNAsi was observed (S7B Fig), indicating that high-AAMRGS subgroup is associated with a poorer prognosis.

To explore the relationship between AAMRGS and intrinsic molecular subtypes, we analyzed the distribution of PAM50 subtypes in the two risk groups. In TCGA cohorts, the low-AAMRGS subgroup exhibited a high percentage of LumA and Normal subtypes, whereas the LumB and Her2 subtypes were predominantly in high-AAMRGS subgroup (Fig 5G). Previous research have demonstrated that LumA exhibits the most favorable prognosis, followed by Normal subtypes, with LumB and Her2 showing less favorable outcomes [54], which is consistent with our result. To assess whether the prognostic value of the AAMRGS was consistent across different molecular subtypes of breast cancer, we conducted Kaplan–Meier survival analyses according to the PAM50 classification. As illustrated in S7C Fig, the AAMRGS effectively discriminated between high- and low-risk patients within each subtypes, supporting its robustness across the intrinsic heterogeneity of breast cancer. In addition, to investigate the functional characteristics of the two risk subgroups, we performed GSVA enrichment analysis. The high-AAMRGS subgroup exhibited upregulation in hallmark pathways including MYC targets, E2F targets, and G2/M checkpoint. KEGG pathway analysis also revealed enhanced activation of several critical pathways in the high-AAMRGS subgroup, including DNA replication, mismatch repair, cell cycle, citrate cycle TCA cycle, and pentose phosphate pathway. Moreover, GOBP enrichment revealed that the control of meiotic spindle assembly, cell cycle, DNA replication initiation, and G2_MI_transition of meiotic cell cycle were upregulated in high-AAMRGS subgroup (Fig 5H). Pathway enrichment suggests that the high-AAMRGS subgroup exhibited enhanced energy metabolism and cell proliferation. These results indicated the high-AAMRGS subgroup was linked with increased clinical aggressiveness, highlighting the biological and clinical relevance of AAMRGS in breast cancer.

### 3.4. The correlation of AAMRGS with genomic variations

**Fig 6:**
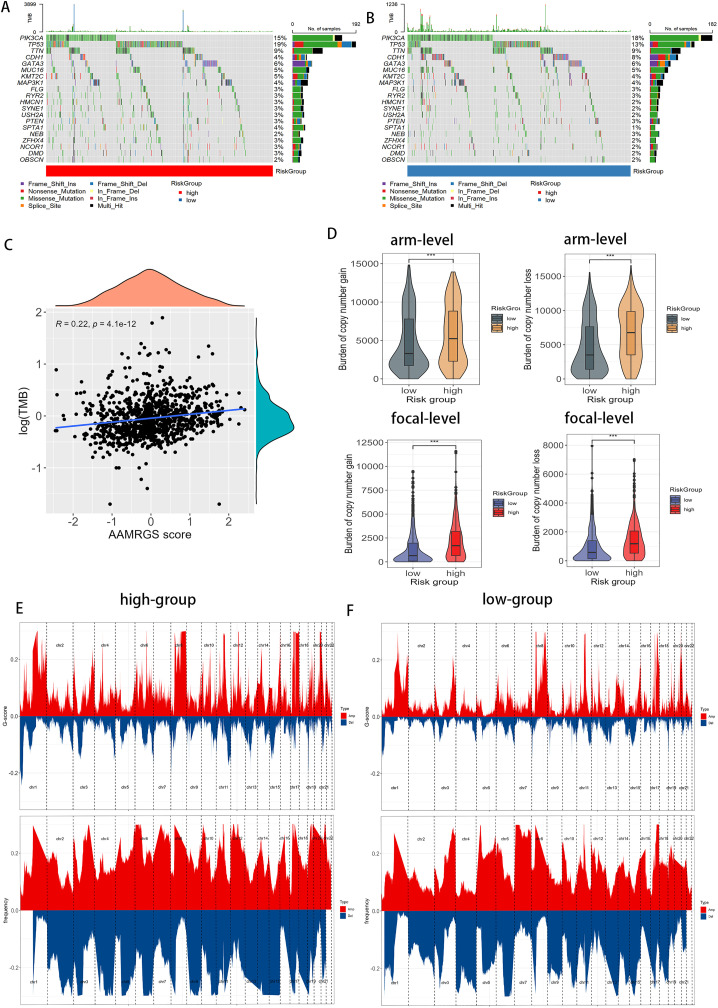
The relationship of AAMRGS with tumor mutations. **(A-B)** The waterfall diagram illustrated the mutation burden status among the top 20 genes across the two risk subgroups. **(C)** The correlation between the TMB and AAMRGS score. **(D)** Copy number variations burden of amplifications or deletions in two AAMRGS-based subgroups in both arm-level and focal level. **(E-F)** The distribution of G-score and altered frequency of chromosomal regions in the high-AAMRGS (E) and low-AAMRGS (F) subgroups. Amplifications are denoted by red, while deletions are represented by blue. Levels of statistical significance (wilcox.test) were defined as NS > 0.05, * p ≤ 0.05, ** p ≤ 0.01, and *** p ≤ 0.001.

To explore the genomic differences between AAMRGS-defined subgroups, we analyzed their mutational landscapes. The high-AAMRGS subgroup exhibited a greater frequency of mutations than the low-AAMRGS subgroup (Fig 6A, 6B). Missense mutations were the predominant type of alteration in both subgroups. *TP53*(19%), *TTN*(9%), *KMT2C*(5%), *MAP3K1*(5%), *HMCN1*(3%), *SYNE1*(3%), *SPTA1*(4%), *ZFHX4*(3%), and *NCOR1*(3%) somatic mutation profiles were more prevalent in the high-AAMRGS subgroup. In contrast, mutations in *PIK3CA*(18%), *CDH1*(8%), and *NEB*(3%) were more frequently in the low-AAMRGS subgroup. We performed spearman correlation analysis to further detect the association between TMB and AAMRGS score, revealing a significant positive correlation. (Fig 6C). Furthermore, we examined the differences in copy number variation (CNV) between subgroups. The high-AAMRGS subgroup displayed a greater CNV burden than the low-AAMRGS subgroup, characterized by more extensive arm-level and focal-level copy number amplifications or deletions (Fig 6D). Consistently, GISTIC analysis revealed that the high-AAMRGS subgroup harbored a greater number of significantly altered genomic regions, including both amplifications and deletions (Fig 6E, 6F). These findings suggested that the high-AAMRGS subgroup is characterized by genomic instability, which may contribute to its poorer clinical outcomes.

### 3.5. The correlation of AAMRGS with immune landscape

**Fig 7:**
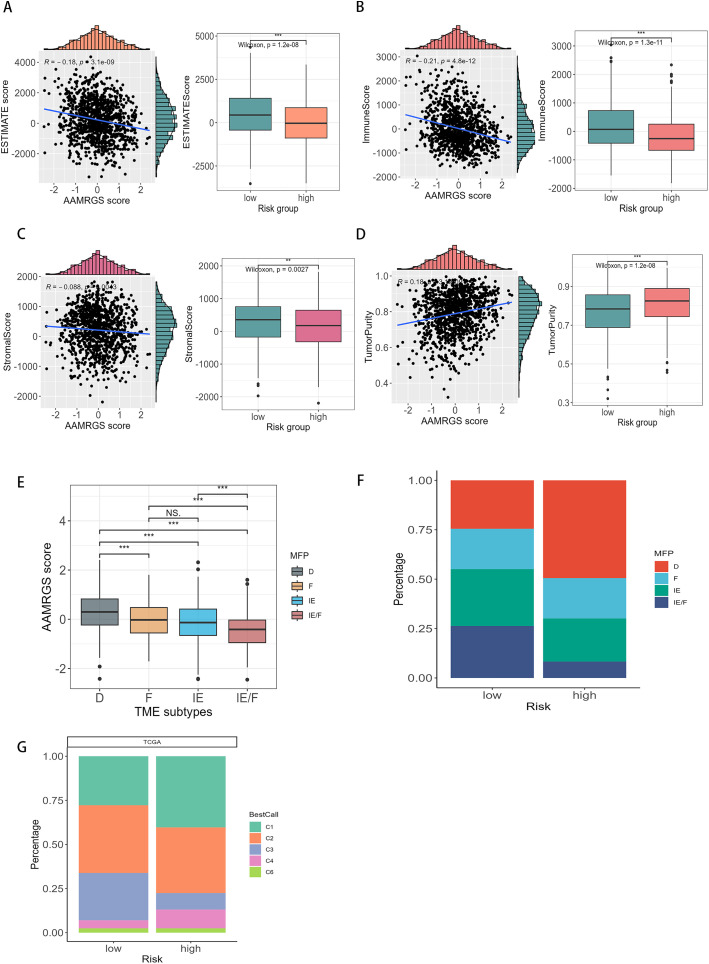
The connection of AAMRGS with tumor microenvironment. **(A-D)** The relationship between the estimate score **(A)**, immune score **(B)**, stroma score **(C)**, and tumor purity **(D)** with AAMRGS score. **(E)** The distribution of AAMRGS score within different tumor microenvironment subgroups. **(F)** Distribution of tumor microenvironment subtypes in two risk subgroups. **(G)** Distribution of different immune subtypes in two risk subgroups. For two-group comparisons (Wilcoxon rank-sum test) and multi-group comparisons (Kruskal-Wallis rank-sum test followed by pairwise Wilcoxon tests with BH adjustment). Levels of statistical significance (wilcox.test) were defined as NS > 0.05, * p ≤ 0.05, ** p ≤ 0.01, and *** p ≤ 0.001.

To explore the connection between tumor microenvironment and AAMRGS subgroups, we evaluated tumor microenvironmental scores. The immune score, estimate score, and stromal score were all inversely associated with AAMRGS score (Fig 7A–7C), while a positive association was observed between the AAMRGS score and tumor purity (Figs 7D, S7D). Thus, the high-AAMRGS subgroup showed a high tumor purity level (Fig 7D). To delve deeper into the connection between AAMRGS and the immune microenvironment, we discovered significant differences in the allocation of AAMRGS among the D, IE/F, and IE subtypes (Fig 7E). Specifically, the low-AAMRGS subgroup showed a higher prevalence of the IE/F subtype and a lower frequency of the D subtype compared with the high-AAMRGS subgroup (Fig 7F). Moreover, we investigated the character of immune subtypes in breast cancer. The patients divided into six immune subtypes^46^, the C1 and C4 subtypes were more predominant in high-AAMRGS subgroup, whereas the C3 subtype was more prevalent in low-AAMRGS subgroup (Fig 7G).

**Fig 8:**
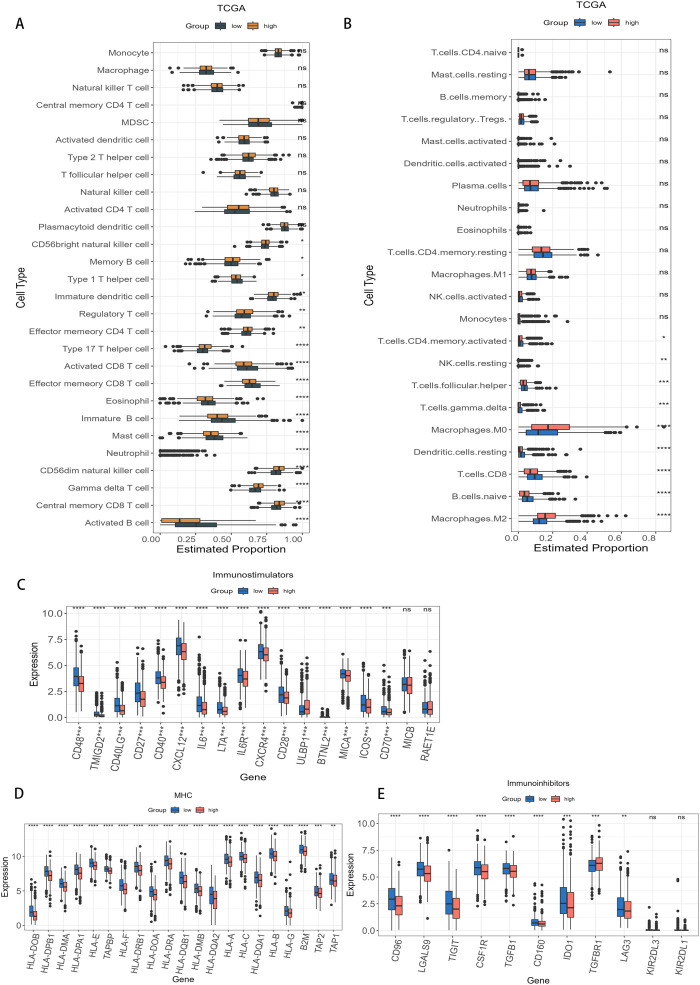
Distribution of immune cell infiltration and immunoregulatory molecules based on AAMRGS. **(A)** The estimated 22 immune cells proportion in two risk subgroups. **(B)** The immune cell proportion in two risk subgroups by CIBERSORT algorithm. **(C-E)** Expression levels of immunostimulators **(C)**, MHC **(D)**, and immunoihibitors (E) in two risk subgroups. Levels of statistical significance (wilcox.test) were defined as ns > 0.05, * p ≤ 0.05, ** p ≤ 0.01, and *** p ≤ 0.001.

To characterize immune infiltration within two subgroups, we calculated the proportion of tumor-infiltrating lymphocytes (TILs) across 28 subpopulations [55]. The high-AAMRGS subgroup exhibited a higher prevalence of type 17 T helper cell, immature dendritic cell, regulatory T cell, CD56dim natural killer cell, effector memory CD4^+^ T cell, activated dendritic cell and central memory CD8^+^ T cell. Conversely, the low-AAMRGS subgroup showed higher abundances in type 1 T helper cell, activated CD8^+^ T cell, immature B cell, mast cell, eosinophil, and activated B cell (Fig 8A). According to the CIBESORT algorithm, the high-AAMRGS subgroup displayed greater levels of Macrophages.M2 and Macrophages.M0, wherease the low-AAMRGS subgroup had a higher level of Macrophages.M1, T.cells.CD8, B.cells.naive, Dendritic.cells.resting, and T.cells.CD4.memory.activated (Fig 8B). Taken together, the low-AAMRGS subgroup displayed a greater level of anti-tumor immune infiltration than high-AAMRGS subgroup. Conversely, the high-AAMRGS subgroup exhibited elevated infiltration of immunosuppressive cells in comparison to low-AAMRGS subgroup.

Beyond immune cells, immunostimulatory factors, major histocompatibility complex (MHC), and immunosuppressive factors are all key components that constitute the immune microenvironment. Therefore, we examined the expression levels of immunostimulators, MHC, and immunoinhibitors and found that most immunostimulators, MHC, and immunoinhibitors were expressed at higher levels in low-AAMRGS subgroup than high-AAMRGS subgroup (Fig 8C–8E). Furthermore, the assessment of anti-tumor immune cycle activity revealed that most immune cycle steps were incrased in the low-AAMRGS subgroup than high-AAMRGS subgroup (S8A Fig). In summary, the low-AAMRGS subgroup appeared to be situated within a hot tumor immune microenvironment.

### 3.6. The correlation of AAMRGS with therapeutic sensitivity

**Fig 9:**
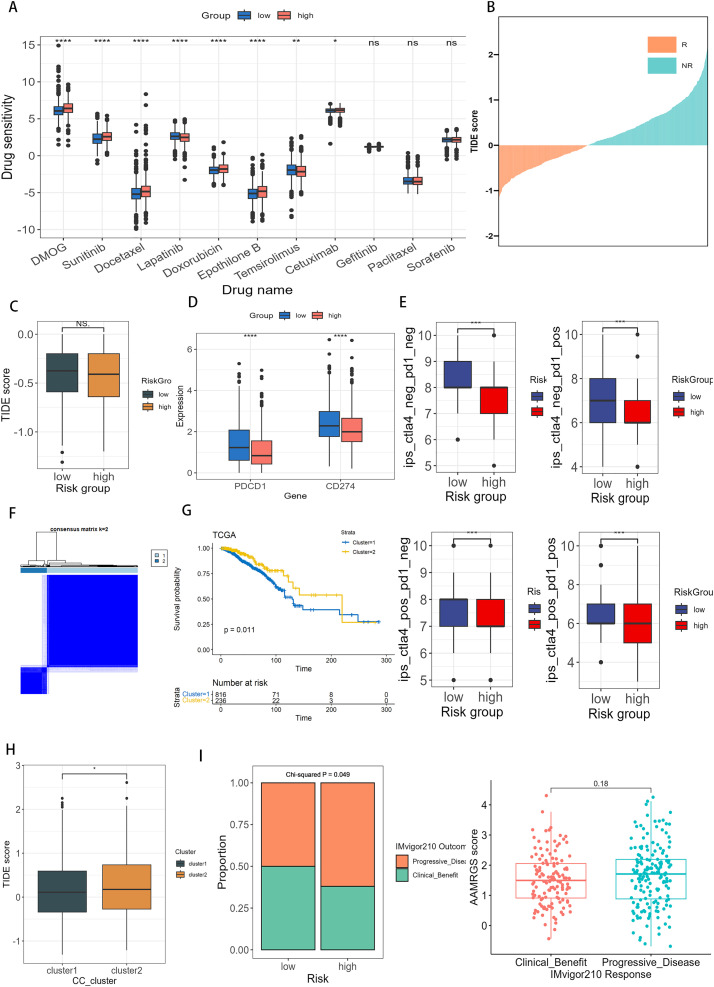
Sensitivity of chemotherapy and immunotherapy based on AAMRGS. **(A)** Associations between AAMRGS score and chemotherapeutic drugs. **(B)** The distribution of non-responder (NR) and responder (R). **(C)** The distribution of TIDE score in two risk subgroups. **(D)** The expression of PDCD1 and CD274 in two risk subgroups. **(E)** The distribution of IPS in two risk subgroups was categorized according to CTLA4 and PD-1. **(F)** Hierarchical Clustering Dendrogram. Two distinct clusters were identified. **(G)** Kaplan–Meier plot of cluster1 and cluster2 subgroups. **(H)** The distribution of TIDE score in cluster1 and cluster2 groups. **(I)** The distribution of response outcomes in two risk subgroups and the distribution of AAMRGS score in response outcome: clinical benefit and progressive disease. For two-group comparisons (Wilcoxon rank-sum test) and multi-group comparisons (Kruskal-Wallis rank-sum test followed by pairwise Wilcoxon tests with BH adjustment). Levels of statistical significance (wilcox.test) were defined as ns > 0.05, * p ≤ 0.05, ** p ≤ 0.01, and *** p ≤ 0.001.

To evaluate the potential of AAMRGS as a biological marker for predicting chemotherapeutic response in breast cancer patients, we estimated the IC50 values [56] of multiple chemotherapeutic drugs between the two risk subgroups. Patients in low-AAMRGS subgroup demonstrated greater response to Lapatinib and Temsirolimus (Fig 9A), whereas patients with high-AAMRGS exhibited greater responsiveness to the DMOG, Sunitinib, Docetaxel, Doxorubicin, Epothilone B, and Cetuximab (Fig 9A). We further analyzed the publicly available GSE25055 chemotherapy cohort to validate these findings. Patients in low-AAMRGS subgroupexhibited higher sensitivity to chemotherapy and better survival outcomes (S8B, S8C Fig), while high-AAMRGS patients showed a greater tendency towards resistance. These findings provide preliminary clinical evidence supporting the predictive value of AAMRGS in chemotherapy response and prognosis. In terms of immunotherapy response, we applied TIDE analysis to obtain the responser and non-responser groups (Fig 9B). The No statistically significant differences were observed between two risk subgroups (Fig 9C). However, the expresssion levels of PDCD1 (PD-1) and CD274 (PD-L1) were higher in low-AAMRGS subgroup (Fig 9D), suggesting a potentially more active immune checkpoint environment. Furthermore, the Immunophenotypic Score (IPS) analysis can also estimate the potential of immunotherapy response especially for ICB treatment. We found that low-AAMRGS patients had higher IPS compared to high-AAMRGS patients, indicating that the low-AAMRGS patients may display a better response to immunotherapy (Fig 9E). To further investigate the response to immunotherapy, we performed consensus clustering based on AAMRGS genes, which divided patients into Cluster 1 and Cluster 2 (Fig 9F). The Cluster 1 had a better survival rate (Fig 9G) and was more sensitive to immunotherapy (Fig 9H). Additionally, in the validation IMvigor210 cohort, we found that there were more clinical benefit patients in the low-AAMRGS subgroup than high-AAMRGS subgroup. Although the AAMRGS score in clinical benefit and progressive disease groups was not significant, the clinical benefit group showed lower than the progressive disease group (Fig 9I). These findings suggest that AAMRGS might be related to chemotherapy and immunotherapy in breast cancer.

### 3.7. Functional analysis of 13 genes and vallidation of key genes expression by RT-qPCR

**Fig 10:**
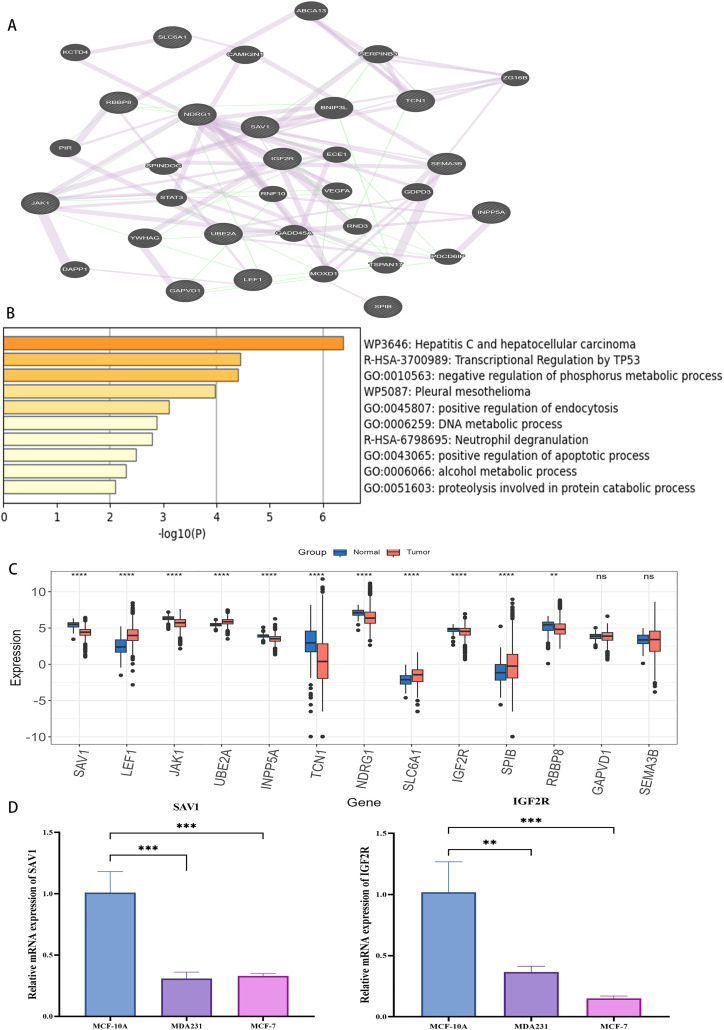
The functional analysis of the co-expression genes related to the 13 hub genes. **(A)** The protein interaction analysis. **(B)** The pathway enrichment analysis. Only top10 terms with were listed. **(C)** The expression of signature genes between tumor and normal samples. **(D)** The mRNA expression levels of *SAV1*, *IGF2R* were measured by RT-qPCR in MCF-10A, MDA-MB-231 and MCF-7 cell lines and normalized to *GAPDH* (p < 0.05). Levels of statistical significance (wilcox.test, one-way ANOVA) were defined as ns > 0.05, * p ≤ 0.05, ** p ≤ 0.01, and *** p ≤ 0.001.

To explore the potential function of the 13 genes, we performed a protein–protein interaction (PPI) analysis using the GeneMANIA. A network consisting of 34 interacting genes was constructed, revealing that hub genes (such as *IGF2R*, *SAV1*) occupied central positions, indicating their potential biological importance in tumorigenesis (Fig 10A). Subsequently, the functional enrichment analysis was performed on the 34 co-expression genes. The results showed that the co-expression genes were primarily mapped into DNA metabolic process, negative regulation of phosphorus metabolic process, proteolysis involved in protein catabolic process, Transcriptional Regulation by *TP53* (Fig 10B). Furthermore, we compared the expression levels of the identified 13 genes between tumor and normal tissues in TCGA (Fig 10C). To further validate the robustness of our prognostic signature, we selected two representative genes (*SAV1*, *IGF2R*) for experimental verification, based on their statistical significance, hazard ratio distribution, and supporting evidence from external dataset (S8D Fig) and HPA database. Quantitative real-time PCR (RT-qPCR) was performed in one normal breast epithelial cell line (MCF10A) and two breast cancer cell lines (MCF7 and MDA-MB-231). As shown in Fig 10D, the expression levels of *SAV1* and *IGF2R* were markedly reduced in breast cancer cells compared to MCF10A. These results were consistent with our bioinformatics analysis, thereby supporting the reliability of the identified prognostic genes.

## 4. Discussion

Metabolic reprogramming is recognized as a hallmark of cancer [11] and has been emerged as an essential therapeutic target for cancer [57,58]. In addition to glycolytic metabolism pathways, amino acid metabolism plays a central role in sustaining cancer cell proliferation [13], and becoming the promising prognostic biomarkers [59–63]. Several prognostic models based on amino acid metabolism–related genes (AAMRGs) have been developed across various cancers, demonstrating their potential of molecular signatures in outcome prediction across malignancies [60,64–67]. However, there is still a lack of systematic understanding of the characteristics of breast cancer associated with AAMRG.

We built an AAMRGS by integrating 98 combinations of 10 machine learning algorithms to predict breast cancer prognosis. A total of 89 genes were identified as being related to survival across the TCGA, METABRIC, and GSE96058 using Kaplan-Meier analysis and univariable cox regression. Among all tested combinations, the Lasso+StepCox[both] model achieved optimal predictive performance and was used to construct the final AAMRGS.To analyze the ability of the AAMRGS for predicting prognosis, ROC and K-M analysis revealed that AAMRGS distinguished two risk groups with good predictive performance. Importantly, validition across large independent datasets (METABRIC, GSE96058, and GSE20685 (S3C–S3E Fig)) confirmed its robustness and generalizability. Compared to previously published amino acid metabolism-related signatures, our AAMRGS showed superior prognostic accuracy and stability (Fig 2B). Moreover, the AAMRGS was performed better than individual clinical factors, underscoring its superior prognostic capability and potential clinical utility.

To enhance clinical applicability, the AAMRGS-based nomogram was created to forecast breast cancer patients’ OS and support the selection of suitable treatment strategies. We integrated AAMRGS with clinical variables to develop AAMRGS-based nomogram with improved performance (AUC 0.80–0.82). Moreover, the nomogram exhibited reliable performance in DCA, calibration, and NRI/IDI analyses, suggesting its feasibility as a clinical decision-support tool. The AAMRGS and the AAMRGS-based nomogram developed in our study have significant implications for clinical decision-making. For instance, patients with high AAMRGS scores may benefit from more aggressive treatment strategies (e.g., adjuvant chemotherapy or targeted therapy), whereas low-score patients may avoid overtreatment. Despite requiring further validation, this nomogram provides a user-friendly approach for personalized survival prediction.Additionally, AAMRGS demonstrated predictive capacity across multiple tumor types (COAD, KIRC, LGG, and SKCM), suggesting its potential as a pan-cancer prognostic tool.

Given the heterogeneity of breast cancer, its prognosis is influenced by many factors including its biological characteristics, patients characteristics, immune microenvironment, and treatments. Our comprehensive analysis revealed that high-AAMRGS subgroup was associated with worse clinical outcomes such as advanced stage, older age, higher mRNA-related stemness index, and more aggressive phenotypes. Functional enrichment analyses indicated that high-AAMRGS subgroup exhibited activation of glycolysis, oxidative phosphorylation, cell cycle, DNA replication, citrate cycle, and pentose phosphate pathway. Furthermore, PAM50 analysis showed enrichment of more malignant subtypes (LumB and HER2-enriched) in the high-AAMRGS subgroup, while low-AAMRGS patients predominantly displayed less aggressive phenotypes. The underlying mechanisms of these poorer phenotypes may be associated with more frequent genomic alterations and we noted a higher CNV burden and TMB in the high-AAMRGS subgroup. Importantly, the AAMRGS effectively stratified survival within PAM50 subtypes and diverse clinical characteristics, indicating its independent prognostic value. These results suggest that AAMRGS has the potential to distinguish the breast cancer patients’ clinical outcomes and could guide clinical decision-making.

Among the thirteen genes contained in AAMRGS, several have been previously implicated in breast cancer progression and prognosis. For example, *JAK1* expression is significantly reduced in breast invasive carcinoma and assocites with prognosis and immune infiltration [68]. *JAK1* also interacts with *DPYSL2* to promote breast cancer cell migration [69]. *SLC6A1* promotes invasion and migration [70], while *SEMA3B* acts as a tumor suppressor by inducing apoptosis [71]. *LEF1* regulates breast cancer cell proliferation [72], and *IGF2R* enhances tumor cell invasion and migration [73] and serves as a poor prognostic marker in triple-negative breast cancer patients [74]. *NDRG1* contributes to tumor aggressiveness by cell proliferation and lipid metabolism regulation [75,76], whereas *GAPVD1* [77], *INPP5A* [78], *TCN1* [79], *SAV1* [80], *RBBP8* [81], *SPIB* [82], and *UBE2A* [83] also exhibit oncogenic or tumor-suppressive properties associated with prognosis. To further explore the biological functions and potential interactions among these genes, we constructed a protein–protein interaction (PPI) network using GeneMANIA, which identified *IGF2R* and *SAV1* occupy central positions within the network, suggesting their regulatory importance in tumorigenesis. Functional enrichment analysis of co-expressed genes indicated significant enrichment in pathways related to DNA metabolic processes, proteolysis, transcriptional regulation by *TP53*, and phosphorus metabolism, highlighting their involvement in genomic stability, cell cycle control, and apoptosis regulation. These biological processes are consistent with the mechanisms through which metabolic reprogramming influences tumor progression, providing further support for the functional coherence of the identified prognostic genes. Given their central roles in the PPI network, statistical significance, and supportive evidence from external databases, we selected *SAV1* and *IGF2R* as representative genes for experimental validation. Experimental validation confirmed that *SAV1* and *IGF2R* were significantly downregulated in breast cancer cell lines, consistent with public datasets, underscoring their biological relevance. Collectively, these findings indicate that AAMRGS signature captures genes that are biologically interconnected and functionally relevant to tumor metabolism, DNA repair, and cellular signaling, underscores the utility of AAMRGS as a robust prognostic signature in breast cancer.

In terms of treatment prediction, precision and personalized treatment is essential for improving clinical outcomes in breast cancer. We observed that the high-AAMRGS subgroup exhibited greater sensitivity to several chemotherapeutic agents, including DMOG, Sunitinib, Docetaxel, Doxorubicin, Epothilone B and Cetuximab. It might be attributed to the enhancement of high-AAMRGS subgroup in DNA replication initiation, cell cycle, and G2_MI_transition of meiotic cell cycle. Then, we explored the ability of AAMRGS for predicting immunotherapeutic sensitivity, immune checkpoint inhibitors were one of the most critical treatments of immunetherapy [84]. The responsiveness of immunotherapy appears to be closely tied to the characteristics of the immune microenvironment. The low-AAMRGS subgroup displayed higher immune scores, enriched extensive infiltration of anti-tumor cells (type 1 T helper cells, CD8 + T cells), upper immune activity of immune cycle, and greater expression of immunostimulators and MHC. Our findings indicated that the low-AAMRGS subgroup may represent a hot tumor microenvironment, which is potentially more responsive to immunotherapy. Activated CD8^+^ T cells were one of the most critical anti-tumor immune cells which had a favorable effect on breast cancer patients’ survival [85]. On the contrary, the high-AAMRGS subgroup exhibited highertumor purity and greater infiltration of immunosuppressive cells such as Macrophages M2 and regulatory T cells, which are correlated with poor survival [86–89]. Moreover, the high-AAMRGS subgroup displayed a higher proportion of D subtype, while the low-AAMRGS subgroup had a greater percentage of IE/F subtype. TMB was also responded to immunotherapy [90]. Our study discovered a positive correlation between the TMB and AAMRGS. However, the ability of high TMB was not effective in predicting immune checkpoint blockade in breast cancer [91]. Therefore, we further examined the immune checkpoints’ expression. The immune checkpoint analysis revealed that PD-L1 (Fig 9D) and other checkpoint molecules (S4B Fig) were significantly upregulated in low-AAMRGS subgroup, suggesting enhanced immune responsiveness. Despite the TIDE analysis revealing no statistical difference in two risk subgroups, the IPS analysis showed enhanced IPS scores in low-AAMRGS subgroup. TIDE primarily assesses T-cell dysfunction and exclusion, which is more predictive in highly inflamed tumors (e.g., melanoma or NSCLC), whereas the IPS quantifies the global immune activation potential, including antigen presentation, effector cell activation, and checkpoint expression. Given the relatively low immune infiltration in breast cancer, the IPS may more accurately reflect the underlying immunogenic potential than TIDE. Consistently, validation using the IMvigor210 immunotherapy cohort demonstrated that patients with clinical benefit had lower AAMRGS scores than non-responders and the low-AAMRGS subgroup had more clinical benefit patients, supporting that the low-AAMRGS subgroup possess stronger immune responsiveness and are more likely to benefit from immune checkpoint inhibitors. Taken together, these results indicate that the low-AAMRGS subgroup may exhibit an immunologically “hotter” phenotype with enhanced immune activation and higher potential sensitivity to immunotherapy. These findings indicate that AAMRGS-based prediction could potentially influence both chemotherapy and immunotherapy responses in breast cancer.

Despite the promising results, there were several restrictions associated with our study. Firstly, relying on public available datasets may introduce selection biases, as these datasets often have specific inclusion criteria and may not fully represent the broader patient population. To address this, we validated our findings across multiple independent datasets, which strengthens the reliability of our results. However, the lack of racial and ethnic diversity in these datasets, which are predominantly composed of patients of European descent, may affect the generalizability of our findings to Asian populations. Given that genomic and transcriptomic variations exist among different populations, further validation in diverse cohorts is warranted. Secondly, clinical treatment factors, including chemotherapy, endocrine therapy, and targeted therapy, were not considered due to incomplete clinical data in public resources, which may influence prognostic assessment. Future research should include Asian-based cohorts and integrate detailed treatment information to further validate the robustness of the model. Thirdly, our findings are based on computational analysis and simple experimental validation in vitro, further vitro and vivo experiments are required to gain a deeper understanding of the molecular mechanisms. Nevertheless, our findings provide a strong rationale for future experimental studies. Finally, the limited availability of large-scale multi-omics clinical datasets poses a challenge; future work integrating diverse omics and detailed treatment data will help refine the biological and clinical interpretability of AAMRGS.

## 5. Conclusion

In our study, we established an amino acid metabolism-related gene signature (AAMRGS) involving thirteen genes selected through an integrated machine learning framework. Comprehensive analyses demonstrated that the proposed AAMRGS could predict the probability of breast cancer survival. Moreover, the AAMRGS effectively stratified patients byclinical outcomes and predicted their sensitivity to immunotherapy and chemotherapy. Overall, our study provided a novel sensitive, and robust prognostic indicator that may pave the path for personalized treatment strategies in breast cancer.

## Supporting information

S1 Fig Forest plots of the prognostic value of 89 amino acid metabolism-related genes across three independent cohorts. (TIF)

S2 Fig Robustness of the 89-gene amino acid metabolism-related signature across three independent cohorts. (TIF)

S3 Fig Development and validation of the AAMRGS risk model based on amino acid metabolism-related genes. (TIF)

S4 Fig Pan-cancer survival and immune checkpoint expression stratified by AAMRGS risk subgroups. (TIF)

S5 Fig Comprehensive evaluation of the AAMRGS-based nomogram for predicting overall survival. (TIF)

S6 Fig Kaplan–Meier survival analysis of AAMRGS subgroups. (TIF)

S7 Fig Analysis of AAMRGS subgroups: stemness index prediction, correlation with mRNAsi and tumor purity, and survival outcomes. (TIF)

S8 Fig Comprehensive analysis of AAMRGS subgroups: immune activity, treatment sensitivity, survival outcomes, and gene expression. (TIF)

S1 Table Amino acid metabolism-related genes from GeneCards. (XLSX)

S2 Table The performance of 98 predictive models in training and testing datasets. (XLSX)

S3 Table The published amino acid metabolism-related gene signature. (XLSX)

S4 Table The R packages and algorithms used in the study. (XLSX)

S5 Table Amino acid metabolism-related genes from GeneCards enriched pathways. (XLSX)

S6 Table The 89 amino acid metabolism-related genes with prognostic value in breast cancer in three cohort. (XLSX)

S7 Table The details of stepCox[both]. (XLSX)

S8 Table The AAMRGS of 13 amino acid metabolism-related genes with prognostic value in breast cancer(TCGA). (XLSX)

S9 Table The comparison of model performance between the combined model and the AAMRGS model using IDI and NRI at different time points. (XLSX)

## Abbreviations

AAMRGS: Amino acid metabolism-related gene signature
AAMRG: Amino acid metabolism-related genes
TCGA: The Cancer Genome Atlas
GEO: Gene Expression Omnibus database
OS: Overall survival
AUC: area under the curve
ROC: receiver operating characteristic
K-M: Kaplan-Meier
GSVA: gene set variation analysis
GO: gene ontology
KEGG: Kyoto Encyclopedia of Genes and Genomes
TMB: tumor mutation burden
IPS: Immunophenoscore.
